# The correlation between corona virus disease 2019 and alopecia areata: a literature review

**DOI:** 10.3389/fimmu.2024.1347311

**Published:** 2024-07-03

**Authors:** Ying Xie, Shuying Lv, Sha Luo, Yuxuan Chen, Meijiao Du, Yonglong Xu, Dingquan Yang

**Affiliations:** ^1^ School of Clinical Medicine, Beijing University of Chinese Medicine, Beijing, China; ^2^ Department of Dermatology, China-Japan Friendship Hospital, Beijing, China; ^3^ Department of Dermatology, The First Affiliated Hospital of Hunan University of Chinese Medicine, Changsha, China

**Keywords:** COVID-19, alopecia areata, vaccination, correlation, pathogenesis, treatment

## Abstract

Corona virus disease 2019(COVID-19) is one of the most serious respiratory pandemic diseases threatening human health for centuries. Alopecia areata (AA) is a sudden patchy hair loss, an autoimmune disease, which seriously affects the image and mental health of patients. Evidence shows that the risk of autoimmune diseases significantly increases after COVID-19, and is positively correlated with the severity, with a significant increase in the risk of alopecia in those over 40 years old. The relationship between COVID-19 and AA has become a hot topic of current research. Strengthening the research on the correlation between COVID-19 and AA can help to identify and protect susceptible populations at an early stage. This article reviews the research progress on the epidemiological background of COVID-19 and AA, the situation and possible mechanisms of AA induced by COVID-19 or COVID-19 vaccination, and potential treatment methods.

## 1 Introduction

Corona virus disease 2019(COVID-19) is a systemic multi-organ injury disease mainly targeting the lungs, caused by severe acute respiratory syndrome coronavirus 2 (SARS-CoV-2). It has strong transmissibility and quickly leads to a global pandemic. To resist the spread of the virus, the number of COVID-19 vaccinations has rapidly increased worldwide, followed by the occurrence of some adverse reactions, one of which is hair loss. As a systemic multi-organ injury disease, COVID-19 has been shown to be closely related to skin diseases (1), with hair loss being one of the most common sequelae of COVID-19. Alopecia areata (AA) and telogen effluvium (TE) are the most common types of hair loss in the context of COVID-19 (2).

AA is a T-cell-mediated autoimmune disease in which hair follicles lose immune privilege, related to genetic, immune, and psychological factors. Depending on the extent of hair involvement, it can be classified into localized or circumscribed alopecia areata, alopecia totalis (AT), and alopecia universalis (AU). The lifetime risk of AA is 2.1%, with a total prevalence of 0.1% to 0.2% (3). Studies estimate that the global incidence of AA ranges from 0.57% to 3.8% (4), with no significant difference in incidence between men and women. Currently, the relationship between COVID-19 and AA is not clear, but there have been many reports of new onset, recurrence, or worsening of AA after SARS-CoV-2 infection or COVID-19 vaccination. This review summarizes the relevant studies on the new onset or recurrence of AA or the exacerbation of existing AA after SARS-CoV-2 infection or COVID-19 vaccination, and deduces the possible mechanisms of inducing AA, to better clarify the association between AA and COVID-19.

## 2 Materials and methods

### 2.1 Methods

This paper explores the association between AA and COVID-19 by searching for relevant literature on new or recurrent AA after COVID-19 or COVID-19 vaccination, or exacerbation of existing AA. The specific method was as follows: CNKI, Wanfang, VIP, and Pubmed were used as data sources, and (COVID-19 OR SARS-CoV-2 OR “Coronavirus Disease-19” OR “Novel coronavirus”) AND (Alopecia Areata OR Hair loss OR Bald spot) were used as the search formula to search papers included before December 2023. Inclusion criteria for literature: Literature that is publicly published, downloadable, and with full text available for reading, reporting patients diagnosed with COVID-19 and AA, or diagnosed with AA after receiving the COVID-19 vaccine. Exclusion criteria for literature: Duplicate reports, literature on case reports not confirmed by diagnosis.

### 2.2 Results

A total of 51 patients reported in 35 literature articles were included.

Cases of new-onset AA after COVID-19 were reported from Italy, Saudi Arabia, Japan, and the United States, accounting for 11 cases (**Table 1**). Most patients were adults, with 5 cases being diffuse AU and another case presenting with a distinctive pattern of AA in the beard area, characterized by well-defined circular bald patches. Only Italy reported 4 child patients, all of whom exhibited patchy hair loss.

**Table 1:** Currently reported cases of new-onset AA following COVID-19.

Gender	Age	Past History	Family History	Hair Color	Onset after COVID-19	COVID-19 Symptoms	Alopecia Situation	Pull Test	Trichoscopy	Treatment	Nationality	Reference
M	8	None	\	Black	7 weeks	Mild	3x4cm patch	+	Exclamation-mark hairs and black dots	Topical corticosteroids	Italy	(5)
M	9	None	\	Blonde	4 weeks	Mild	3x3cm patch	+	Exclamation-mark hairs and black dots	Topical corticosteroids	Italy	(5)
F	8	None	\	Brown	3 weeks	cough, fatigue, general malaise, fever	2x2cm patch	+	Exclamation-mark hairs, black dots, centrally vellus hair	Topical corticosteroids	Italy	(5)
M	13	None	\	Brown	4 weeks	Multisystem inflammatory syndrome	1cm patch	–	\	\	Italy	(5)
M	38	None	None	Brown	1 month	\	Well-defined patch in the beard	\	Yellow dots, black dots, broken hair, vellus hair	Niacin, resorcinol, and hyaluronic acid three-color lotion	Italy	(6)
F	29	None	Brother has Bruton’s syndrome	\	1 month	Fever and dry cough	AU	+	Black dots, yellow dots, exclamation mark hair, and regenerating hair	Triamcinolone acetonide, corticosteroids, and bimatoprost.	Italy	(7)
F	20	None	None	Black	\	Loss of smell, headache, and sore throat	AU, nail dystrophy	\	\	Tofacitinib	Saudi Arabia	(8)
F	47	\	\	Black	3 weeks	Severe	Diffuse AA	+	Black dots, yellow dots	\	Japan	(9)
F	28	Allergic rhinitis	Father with psoriatic arthritis	Brown	1 month	Mild	AU	\	Yellow dots, vellus hair, black dots, exclamation-mark hairs	Intralesional triamcinolone, methylprednisolone, PRP	USA	(10)
F	56	\	\	\	\	\	AU	\	\	\	USA	(11)
M	28	\	None	\	1 month	Headache and sore throat	Two patches	\	\	aPRF	USA	(12)

Lebanon, Saudi Arabia, and Brazil collectively reported on the post-COVID-19 changes in condition for 4 AA patients (**Table 2**). Among these, the condition did not deteriorate for 3 patients after COVID-19; rather, it continued to improve. The remaining patient did not experience a recurrence of AA following COVID-19 but instead developed TE.

**Table 2:** Reported cases of alopecia patients after COVID-19.

Gender	Age	Duration of Illness	Family History	Alopecia Situation Before COVID-19	Hair Color	COVID-19 Symptoms	Changes in Condition After COVID-19	Treatment and Effect After COVID-19	Nationality	Reference
F	32	13 years	\	AT	Black	Mild	3 months after recovery, the hair fully regrew.	Continued discontinuation of tofacitinib.	Lebanon	(13)
F	27	16 years	\	AU	Black	Mild	Continued improvement during COVID-19	Tofacitinib	Saudi Arabia	(14)
F	33	23 years	\	AU	\	Mild	No changes in AA	Tofacitinib	Saudi Arabia	(14)
F	24	\	\	Hair fully regrew after treatment	Brown	Loss of smell, deafness	Telogen effluvium after COVID-19	Tofacitinib; no effect on telogen effluvium	Brazil	(15)

Cases of AA after COVID-19 vaccination have been reported more frequently in the United States and Italy, and also in Egypt, Brazil, Uruguay, Qatar, Japan, Iran, and Taiwan, China (**Table 3**). Most patients are adults, with only one pediatric patient in Italy, and the majority of patients have received the Pfizer/BioNTech vaccine, followed by AstraZeneca and Moderna.

**Table 3:** Reported cases of alopecia after COVID-19 vaccination.

Gender	Age	Past History	Family History	Vaccine Received	The number of vaccinations that induce AA	Time of Onset After Vaccination	Alopecia Situation	Pull Test	Trichoscopy	Treatment	Nationality	Reference
F	32	AA	None	AstraZeneca	\	A few days	Localized patchy AA	\	Black dots, broken hairs, regrowth hairs, exclamation-mark hairs	\	Egypt	(16)
F	27	None	None	BioNTech/Pfizer	vaccine booster	15 days	Diffuse AA	\	Yellow dots, black dots, dystrophic hairs, and white hairs	\	Brazil	(17)
F	27	Polycystic ovary syndrome and AU	None	Pfizer,SinoVac,AstraZeneca	AstraZeneca booster	8 days	AU	\	\	Topical dexamethasone and clobetasol propionate	Ecuador	(18)
M	59	Hypertension and COVID-19	None	Pfizer,SinoVac,AstraZeneca	AstraZeneca booster	17 days	AU	\	\	Topical dexamethasone and clobetasol propionate	Ecuador	(18)
F	51	None	None	AstraZeneca	\	3 days	AT	\	Broken hairs, yellow dots, exclamation-mark hairs	Clobetasol propionate and intralesional triamcinolone	Afro-Caribbean	(19)
F	63	Hypothyroidism, prediabetes, beta thalassemia, and AA	None	Pfizer	first	Within two weeks	From localized patchy AA to AU	\	\	\	Qatar	(20)
F	61	None	\	Pfizer	second	1 week	Localized patchy AA	\	\	Fluconazole, minoxidil solution, and tacrolimus.	USA	(21)
M	15	None	Grandmother and sister have thyroid disease	Pfizer	second	Within 1 week	Localized patchy AA	\	\	Intralesional corticosteroid	USA	(22)
M	16	None	None	Pfizer	first	1-2 weeks	SALT 70,	\	\	Intralesional injection of corticosteroids, and tofacitinib.	USA	(22)
M	22	Elevated thyroid antibodies, normal thyroid function tests	None	Moderna	second	1 month	Patchy AA extending to the beard	\	\	Intralesional injection of corticosteroidst, and tofacitinib.	USA	(22)
M	27	\	\	BioNTech/Pfizer	second	2 weeks	SALT 34 to SALT 59	\	\	\	USA	(23)
F	28	AA and Hashimoto’s thyroiditis	None	Pfizer	second	Within 1 week	AU	\	\	Intralesional injection of corticosteroids, PRP and tofacitinib.	USA	(22)
F	29	The levels of thyroglobulin antibody and thyroid peroxidase antibody are elevated	None	Pfizer	second	Within 1 week	Localized patchy AA	\	\	Intralesional injection of corticosteroids	USA	(22)
M	32	\	\	Moderna	first	2 weeks	SALT 70	\	\	\	USA	(23)
F	33	Fatty liver degeneration, Chronic hepatitis B virus	Brother with AA	Moderna	second	2 months	A large area AA of the scalp	\	\	Intralesional injection of corticosteroids, pimecrolimus, clobetasol and tofacitinib.	USA	(22)
F	33	\	\	BioNTech/Pfizer	first	2 weeks	SALT 99	\	\	\	USA	(23)
M	44	None	None	Moderna	first	2 weeks	From localized patchy AA to AU	\	\	Oral prednisone and topical betamethasone valerate lotion	USA	(24)
F	57	AA	None	Pfizer	second	4 months	Diffuse AA	\	\	tofacitinib	USA	(22)
M	61	None	None	Pfizer	first	2 weeks	AU	\	\	\	USA	(22)
F	62	AA	None	Moderna	second	2 months	AU	\	\	Tofacitinib and bimatoprost eye drops	USA	(22)
F	forties	None	None	Moderna	first	1 week	AU	\	\	Oral prednisone, excimer light, oral cephalosporin and ammonium glycyrrhizinate	Japan	(25)
F	23	None	None	AstraZeneca	first	1 week	AT	\	\	Betamethasone, pimecrolimus cream and oral prednisolone	Iran	(26)
F	26	AA	\	AstraZeneca	second	2 weeks	From localized patchy AA to AU	\	\	Oral prednisolone	Iran	(26)
F	7	None	\	Pfizer	second	20 days	Localized patchy AA	\	\	\	Italy	(27)
M	18	None	None	BioNTech/Pfizer	first	20 days	AT	+	Black dots, vellus hair, and exclamation-mark hairs	Topical high-potency steroids	Italy	(28)
F	23	AA	\	Moderna	first	2 weeks	S2	\	Black dots, broken hairs, yellow dots	\	Italy	(29)
F	25	AA, remission for 4 years	\	Moderna	first	1 week	S3	\	Black dots, broken hairs, yellow dots	\	Italy	(29)
M	31	AA, remission for 2 years	\	Pfizer	first	3 weeks	S1	\	Black dots, yellow dots	\	Italy	(29)
F	32	AA, remission for 2 years	\	Pfizer	first	2 weeks	S3	\	Black dots, broken hairs, yellow dots	\	Italy	(30)
M	42	AA, atopic dermatitis, and celiac disease	\	BioNTech/Pfizer	second	\	AT	\	Yellow dots	Topical high-potency steroids	Italy	(28)
F	51	AA, remission for 1 year	\	Moderna	first	2 weeks	S1	\	Black dots	\	Italy	(29)
F	26	AA	\	Pfizer	second	2 weeks	AU	\	\	Pulse steroid therapy	Taiwan, China	(31)
M	29	AA	\	AstraZeneca	second	1 week	Diffuse AA	\	\	Pulse steroid therapy	Taiwan, China	(31)
M	31	None	None	Pfizer	second	2 days	Localized patchy AA, extending to the beard	+	Yellow dots, black dots, malnourished hairs, and vellus hair	\	Taiwan, China	
F	34	None	\	\	second	\	AT	+	\	Oral prednisolone, PRP	Taiwan, China	(32)
M	42	\	\	AstraZeneca	first	3 weeks	Localized patchy AA	\	Yellow dots, black dots, vellus hair, Exclamation-mark hairs, and tapered hairs	Intralesional triamcinolone acetonide injection	Taiwan, China	(30)

## 3 Discussion

### 3.1 The interplay between COVID-19 and AA

#### 3.1.1 COVID-19 patients seem to be prone to AA

Since the start of the COVID-19 pandemic, the incidence of AA has significantly increased, suggesting that new onset AA may be a dermatological manifestation of SARS-CoV-2 infection (33). A study in Turkey found that the number of AA cases in dermatology outpatient clinics increased after the pandemic, affecting a wide age range of patients. Still, most were adults with no personal or family history of AA (34). A large retrospective cohort study in Korea showed that the risk of autoimmune diseases significantly increased after COVID-19 and was positively correlated with severity, with patients over 40 years old having a significantly increased risk of AA and total alopecia (35). In addition, Rinaldi et al. found that the recurrence rate of AA increased after COVID-19 through a large-scale questionnaire survey (36). This article includes 11 cases of patients who developed new-onset AA after COVID-19, with an average time to onset of about 4 weeks post-infection.

However, there is some disagreement in the research on the relationship between COVID-19 and AA. Rudnicka et al. assessed the relationship between COVID-19 and the severity of existing AA and found that AA symptoms did not significantly worsen after COVID-19, especially in patients who were receiving treatment for AA, thus proposing that mild to moderate COVID-19 is not associated with worsening AA (37). In line with the above findings, among 4 AA patients who contracted COVID-19, there was no significant exacerbation of AA symptoms post-infection.

#### 3.1.2 AA patients may be less susceptible to COVID-19

Research has shown that IFN-γ can lead to the downregulation of ACE2, the receptor for SARS-CoV. Based on this, some scholars have proposed that the elevated levels of IFN-γ in the serum of AA patients may have some role in combating SARS-CoV-2 infection, and this has been confirmed by large-scale cohort studies showing that the risk of COVID-19 is lower in AA patients than in non-AA individuals (38).

### 3.2 The association between COVID-19 vaccination and AA is controversial

Throughout human history, the development of vaccines has greatly changed the course of disease and human societal development. Usually, the development of a new vaccine takes several decades, but due to the urgency of the SARS-CoV-2 pandemic, the process of developing and administering the COVID-19 vaccine has been greatly accelerated. As a large number of people around the world are being vaccinated, reports of adverse events are also very common (18). This article compiles 36 cases of AA following COVID-19 vaccination that have been reported both domestically and internationally.

However, a nationwide population-based study in Korea found that the incidence of autoimmune connective tissue diseases such as AA, psoriasis, and vitiligo did not significantly increase after COVID-19 vaccination. The study pointed out that COVID-19 vaccination does not increase the risk of most autoimmune diseases, including AA (39). Subsequently, another retrospective cohort study in the country came to a consistent conclusion, that the risk of autoimmune diseases did not increase after vaccination, and that vaccination has potential protective effects against the development of COVID-19-related diseases (35).

### 3.3 Pathogenesis

#### 3.3.1 Pathogenesis of AA

The current mechanism of AA is not clear. Still, it is generally believed to be related to the imbalance of various immune genes, immune cells, and immune molecules, leading to the destruction of hair follicle immune privilege. Abnormal immune genes are the basis for the occurrence of AA. Genome-wide association studies have found 139 single nucleotide polymorphism sites significantly related to AA patients, including genomic regions that control immune cell phenotypes, such as Treg cells, human leukocyte antigen (HLA), UL16 binding protein (ULBP), etc (40).

The destruction of hair follicle immune privilege is an important link in the pathogenesis of AA. Local micro-trauma, neurological and psychological factors, and infections can all cause the release of cytokines such as interferon-γ (IFN-γ) and tumor necrosis factor-α (TNF-α), thereby destroying the immune privilege state of the hair follicles. This process involves the mediation of various immune cells, including activated natural killer group 2D (NKG 2D^+^) and cytotoxic T cells (CD 8^+^) (40, 41).

Genetics plays an important role in AA, with 10% to 20% of AA patients having a family history of the disease. The affected genes are related to immune and inflammatory response genes, such as MHC, CTLA 4, and PRDX 5 (18). In addition, there is a high correlation between AA and other immune-mediated diseases (such as thyroiditis, diabetes, and vitiligo), which further supports the theory of immune pathogenesis in AA (42).

Scientists speculate that the autoimmune attack on hair follicles may also be related to viral infections. Viral infections (such as COVID-19) may induce oxidative stress, leading to upregulation of major histocompatibility complex class I (MHC-I) ligands on hair follicles, further activating T cells, destroying hair follicle cells, and releasing IFN-γ and TNF-α around hair follicles, thereby causing a vicious cycle of inflammation (42).

Psychological stress has also gradually become an important cause of AA. Studies have found that the incidence of life stress events before the onset of AA is significantly higher than in normal people, and people with tendencies towards anxiety, depression, and mental stress are also more likely to develop AA (43).

In summary, the development of AA is the result of multiple factors. Genetic factors, hypersensitivity, autoimmunity, stress, trace element deficiency, hormonal changes, vaccination, and infections are all related to the evolution of AA.

#### 3.3.2 Potential association mechanism between COVID-19 and AA

COVID-19 is a disease associated with systemic immune activation caused by SARS-CoV-2. SARS-CoV-2 has an envelope structure, covered with spike proteins, which infect host cells by binding to the angiotensin-converting enzyme 2 (ACE2) receptor on the surface of host cells. The immune response to COVID-19 is multi-layered, and there is a complex interaction between SARS-CoV-2 infection and the host immune system.

At present, the exact mechanism by which COVID-19 induces alopecia is not clear, but based on the pathophysiology of SARS-CoV-2 and alopecia, some possible mechanisms can be speculated. Research suggests that SARS-CoV-2 infection may be related to autoimmunity, and widely distributed tissue antigens may be targets for cross-reactive antibodies directed against SARS-CoV-2 epitopes (44). COVID-19 may trigger an overactivation of the immune system leading to a cytokine storm and inflammatory response. It has been reported that patients with COVID-19 have lower complement levels and positive autoantibodies (45, 46), while plasma concentrations of pro-inflammatory cytokines such as IFN-γ, TNF-α, IL-6, IL-1, IL-2, and IL-17 are significantly elevated, among which IL-6 is believed to be associated with the inhibition of hair shaft elongation and matrix cell proliferation (47, 48). The increase in these pro-inflammatory cytokines can lead to exacerbated inflammation, impairing the immunotolerance of hair follicles and resulting in autoimmune attacks where immune cells mistakenly target hair follicles or hair papilla cells, thereby inducing alopecia.

In addition, dysfunctional ACE2 and its variants may be one of the reasons for imbalance in the inflammatory microenvironment (49, 50). ACE2 is not only the functional receptor for COVID-19 but also an important endogenous antagonist of the renin-angiotensin system (RAS), which plays a key role in maintaining blood pressure and cardiovascular function. Some studies suggest that RAS also plays a significant role in inflammation and autoimmune processes, and an imbalance in RAS may lead to excessive inflammatory responses (51). Research has found that serum ACE levels are higher in more severe cases of AA, while ACE levels in alopecia tissue are significantly lower than in controls. Angiotensin I may play a role in the inflammation of AA, leading to ACE consumption and reduced tissue levels of this enzyme (52).

Simultaneously, activation of the coagulation cascade can lead to microthrombosis formation and blood vessel obstruction in hair follicles, which may also contribute to the development of alopecia. Furthermore, psychological burdens caused by COVID-19, such as mental stress, strict control measures, social restrictions, and unemployment, may also have significant impacts on the development of alopecia (53).

Given the larger proportion of female patients in hair loss caused by COVID-19, female hormones such as estrogen and progesterone may play important roles. Estrogen and progesterone have immunoregulatory and anti-inflammatory effects and can inhibit the release of pro-inflammatory cytokines. Moreover, estrogen and progesterone have protective effects on hair follicles. Estradiol can regulate hair follicle growth and hair cycle through its receptor, while progesterone can reduce the conversion of testosterone to dihydrotestosterone. Dihydrotestosterone is an active form of testosterone, and its increase may lead to hair loss. Therefore, it can be speculated that the acute damage caused by viral infection leads to a significant reduction in systemic estrogen and progesterone levels in female patients, making female COVID-19 patients more susceptible to AA (54).

In summary, the induction of AA by COVID-19 may be a multifactorial process, including abnormal immune responses, the influence of cytokines, vascular dysfunction, and genetic, psychological, and endocrine factors, etc. Further research and clinical observation are needed to clarify the exact roles of these mechanisms in COVID-19-induced AA.

#### 3.3.3 Potential mechanisms by which COVID-19 vaccination could trigger AA

The possible mechanism for the induction of AA by COVID-19 vaccination is the molecular mimicry phenomenon caused by the induction of spike proteins. The COVID-19 mRNA vaccine delivers an mRNA that encodes a protein identical to the spike protein of SARS-CoV-2, thereby inducing the production of immunogenic spike proteins and triggering T-cell and B-cell adaptive immune responses (55). The antibody-mediated response caused by vaccination may cross-react with self-antigens, leading to autoimmunity. These mRNA vaccines continuously produce specific antibodies, including complement products, anti-platelet factor 4, and polyethylene glycol, thereby stimulating the immune system to switch to a chronic inflammatory state.

Research has found that, compared to a control group, serum levels of PARC/CCL18 and GNLY (granulysin), as well as IgG levels against MX1 and METTL3, are elevated in patients with AA related to the COVID-19 vaccine. Among these, PARC/CCL18 promotes the recruitment of CD4^+^Th2 cells to the site of lesions (56), while GNLY is a key mediator released by CD8^+^CTLs, responsible for severe drug-induced delayed hypersensitivity reactions (57). MX1 belongs to interferon-induced GTP-binding proteins (58), and METTL3 plays a role in NK cell activation (59).

Additionally, polyethylene glycol 2000 (PEG 2000) and polysorbate 80, present in mRNA COVID-19 vaccines and the AZD1222/MVCCOV1901 vaccine, are major allergens. T cells from patients with AA associated with mRNA COVID-19 vaccines are significantly activated by PEG 2000 or spike protein, whereas those associated with the AstraZeneca COVID-19 vaccine are activated by polysorbate 80 or spike protein (60).

In summary, post-vaccination with the COVID-19 vaccine, patients may develop AA through an immune response mediated by CD4^+^Th2 and CD8^+^Tc2 cells, triggered by vaccine components or spike protein. CD8^+^CTLs may migrate to hair follicle sites and produce cytotoxic proteins like GNLY, leading to apoptosis of hair follicle cells and ultimately causing AA. The production of autoreactive antibodies and eosinophils by CD4^+^Th2 cells may also be involved in the immunopathological mechanisms of AA related to the COVID-19 vaccine.

Furthermore, studies have found that autoantibodies such as ANA (antinuclear antibodies), IgG-anti-MX1, and IgG-anti-METTL3 are elevated in patients with autoimmune diseases related to the COVID-19 vaccine (60). However, a direct link between AA induced by COVID-19 or vaccination and autoantibodies like anti-spike protein has not yet been established. More research is needed to elucidate this potential association and the underlying mechanisms.

### 3.4 Treatment

#### 3.4.1 Treatment for AA caused by COVID-19

Although hair loss caused by COVID-19 is mostly reversible, patients may need a considerable amount of time to recover from it (14). Therefore, it is particularly important to propose specific treatment and care plans for patients with COVID-19 combined with AA.

Western medicine treatments for AA mainly include oral medications such as corticosteroids and immunosuppressants, and topical medications include biological response modifiers and immunostimulants. Some doctors have suggested that immunosuppressive drugs, including JAK inhibitors like tofacitinib, may alter the immune response leading to a worsening of the infection process, and therefore recommend discontinuing these drugs during a COVID-19 (61). However, studies investigating the impact of JAK inhibitors like tofacitinib on the outcome of COVID-19 have found no statistically significant differences in hospitalization rates, ICU admission rates, or severe infections between patients treated with JAK inhibitors like tofacitinib and other patients (61, 62). A recently published systematic review clarified the potential therapeutic effects of JAK inhibitors and type I IFN on treating COVID-19 and confirmed that they can increase the discharge rate of COVID-19, and reduce ICU admission rates and mortality (63). Therefore, JAK inhibitors may be a potential method for treating COVID-19, by disrupting AP2-associated protein kinase 1 (AAK1) signaling to interrupt the integration of the virus with host cells (64).

In addition, as female hormones such as estrogen and progesterone may play important roles in COVID-19, current research is exploring the possibility of using estrogen and progesterone to treat COVID-19 (65). Some researchers suggest that ACE2 inhibitors could benefit autoimmune patients who are more susceptible to infection by preventing organ damage (66). However the use of ACE2 inhibitors is still challenging, and corticosteroid administration in autoimmune diseases can make it more difficult to diagnose and treat COVID-19 because ACE2 inhibitors prevent fever during the disease.

#### 3.4.2 Treatment for alopecia areata caused by COVID-19 vaccination

Since 1984, there have been reports of hair loss following routine immunizations, with vaccine antigens and adjuvants being considered potential triggers for T-cell-mediated immune responses. Topical corticosteroids, intralesional corticosteroid injections, and topical immunotherapy are the main treatments for AA triggered by COVID-19. A study in South Korea reported a recurrence rate of 27.6% in patients with severe AA who received pulse steroid therapy, with all recurrent patients achieving complete remission after repeated pulse steroid therapy (67). However, patients who relapsed after the COVID-19 vaccine continued to show disease progression despite repeated pulse steroid therapy. It is speculated that elevated levels of vaccine-induced INF-γ may promote the outbreak of T-cell-mediated immune responses, leading to immune dysregulation in susceptible individuals and resulting in treatment-resistant AA (31).

There are reports of patients completely recovering from severe AA using PRP (platelet-rich plasma) therapy, which is more convenient and less toxic than other treatments (32). In addition, studies have shown that tofacitinib and baricitinib can successfully treat AA related to the COVID-19 vaccine (60). However, larger sample size studies are still needed to assess the efficacy of JAK inhibitors in treating AA related to the COVID-19 vaccine.

## 4 Conclusion

Currently, there is not enough evidence to prove a direct relationship between COVID-19 and an increase in the incidence of AA, but studies have found that COVID-19 patients seem to be more prone to trigger AA, while AA patients may be less likely to contract COVID-19. The induction of AA by SARS-CoV-2 infection or COVID-19 vaccination may be a multi-factorial process, including abnormal immune responses, the influence of cytokines, vascular dysfunction, genetic, psychological, and endocrine factors, etc. JAK inhibitors, estrogen, and progesterone may be potential treatments for COVID-19. Further research into the possible pathogenic mechanisms of COVID-19 and AA is of great significance for the prevention, diagnosis, treatment, and prognosis of susceptible populations.

## Author contributions

YX: Writing – original draft. SL: Methodology, Supervision, Writing – original draft. SL: Data curation, Formal analysis, Writing – original draft. YC: Data curation, Formal analysis, Writing – original draft. MD: Data curation, Formal analysis, Writing – original draft. YX: Data curation, Formal analysis, Writing – original draft. DY: Writing – review & editing.
